# A Challenge to Healthy Aging: Limited Social Participation in Old Age

**DOI:** 10.14336/AD.2021.02018

**Published:** 2021-10-01

**Authors:** Angela YM Leung, Alex Molassiotis, Diomedes A Carino

**Affiliations:** ^1^WHO Collaborating Centre for Community Health Services, School of Nursing, The Hong Kong Polytechnic University, Hong Kong SAR.; ^2^Philippine Genome Center, University of the Philippines, Manila, The Philippines

**To the Editor**

There are many challenges in old age as older adults strive for good health, to meet their financial needs, maintain independency and adapt to changes in roles after retirement within different cultural contexts, socioeconomic disparities or access to care [1]. With advancing age, people may find it increasingly difficult to maintain existing and initiate new relationships, as explicated by the disengagement theory of aging [2], and this can result in limited social participation, as the preference may be on maintaining existing social routines rather than expanding social activities [3]. Older adults may lack the opportunity to engage in social activities due to diminished physical functionality and limited access to transportation, and experiencing loneliness and isolation, which in turn can lead to increasing cardiovascular risk, stroke, mental health problems (such as depression and even suicide) and cognitive disorders [4,5]. The impact of limited social participation on health can be detrimental, thus social participation has been identified as an important social determinant of health.

## Illustrative examples of barriers to social participation in the Philippines and Hong Kong

Based on interviews we have carried out for a study with older adults, a 54-year-old female Filipino expressed that she wanted to be involved with a community program for farmers’ wives in her village, but she was reluctant to do so due to poor health status. This made her feel useless and lonely. This is a common situation in the Philippines in which family members ‘restrict’ their older family member to participate in community activities because they consider ‘being inactive’ as ‘safe’. Such belief in the Pilipino culture illustrates a good example of disengagement theory of aging [2], which is assuming that older adults should be limiting their social participation.

Another example is from Hong Kong: a 78-year-old widow mentioned that she did not talk to people in most of the days. She stayed at home, watching television or listening to radio, having no intention to communicate with friends or even adult children. From her perspective, ‘talking to people’ means ‘bothering others’ and she did not want to bother others as she perceived people are very busy for their own lives. This is a typical cultural and personal value among Chinese people who do not bother to seek help even though they may be sick [6], as familial harmony is of great importance. Although these older persons did not indicate their loneliness or considered themselves as ‘being socially isolated’, observation should continuously be made for any signs of depression or cognitive decline in such cases.

## Supporting Social Participation in Old Age

Cultivating an environment conducive of social participation in old age needs the collaboration of many parties in different sectors, counting on family members, neighbors, the social sector, health care services and public facilities like transportation [7]. First of all, building up relationships with friends, family, and neighbors can enhance community participation, as this enables and motivates individuals to access their community resources. Maintaining connections through routine dinners, visits, baby-sitting, participating in festival events, or greetings through phone or internet contacts should be encouraged. Neighbors with similar age can provide peer support, by arranging activities together within their neighborhood. One-to-one interventions, e.g., befriending, or addressing maladaptive social cognitions, have been reported to be positively affecting social connection via reduced loneliness [8]. Reminiscence therapy is particularly helpful to reduce social isolation [9], which is one of the social connection components [10]. Another strategy to promote social participation is to maintain or extend older adults’ prior interests. Like-minded people can communicate well, and they would find the activities meaningful and enjoyable, and maintain relationships. The third strategy is to increase older people’s knowledge and awareness of their own health. The objective of improving one’s own health is to make older adults connect with health service providers in the local area, forging the habit of participating in health-related activities and making them stay tuned with resources in their community. Furthermore, resources and environment (including culture) should be drawn to support social participation. Transportation and age-friendly physical and social environments (that are accessible, equitable, inclusive, safe and secure, and supportive) are essential to enable social participation. Taking an example from Hong Kong, all older adults are entitled to a discounted fee (HK$2=US$0.26) for travelling in public transportation, and this highly encourages older adults to make a visit to their friends and relatives who are living in other districts or join leisure activities out of their neighborhood (www.scmp.com/comment/letters/article/3108357/why-hong-kong-needs-speed-action-hk2-transport-elderly). Jo Cox Commission on Loneliness in the United Kingdom further highlighted the public health implications of loneliness, creating a national dialogue that led to the appointment of a minister of loneliness, which can be regarded as society level intervention to improve social connection [11].

A more recent trend is that of social prescribing as a strategy to direct socially isolated older adults to community-based services through a referral system, addressing the social determinants of health that contribute to poor health [12,13]. Social prescribing starts in primary care settings in which doctors refer older adults to ‘link workers’ who are familiar with the community resources and support older adults to identify social, economic or environmental problems that affect their physical and psychological health, and in this way enhancing social capital and allowing older adults to manage their own well-being [12]. Social prescribing is being widely implemented in the United Kingdom, but its potential efficacy and cost effectiveness warrants further investigations [14].

Last year has seen an added complication to efforts in promoting social participation in older adults due to COVID-19. Social isolation and loneliness have increased in this vulnerable population due to social distancing measures against COVID-19, and action plans to enhance social participation in older adults need to be more flexible and adjust to the realities that this global pandemic has introduced in the society [15,16].

## Social participation and WHO Healthy Aging Framework

WHO advocated for Healthy Aging and developed an action plan for the next decade (Decade of Healthy Aging 2020 -2030) (www.who.int/initiatives/decade-of-healthy-aging). Within this plan, the value of social participation, with emphasis on community-based activities and interpersonal interactions, stand out, while resource sharing, and multi-sector collaboration are encouraged to cultivate a suitable environment for older people to engage in activities in the community and sustain their independency as they age. The WHO action plan, which was also proclaimed by the United Nations as the Decade of Healthy Aging (resolution 75/131), clearly establishes of foremost importance the creation of environments and opportunities that enable people to be and do what they value throughout their lives.

The value of social participation, with emphasis on community-based activities and interpersonal interactions, are important to consider in the effort of enhancing healthy aging, while resource sharing, and multi-sector collaboration should be encouraged to cultivate a suitable environment for older adults to engage in activities in the community and sustain their independency as they age. Policy is needed to support social participation in old age for all countries, as we can see the benefits of social participation brought to individuals, families and societies [17]. Notwithstanding the role of social participation in improving health in older adults, the importance of respecting solitude activities that may provide well-being, particularly in the oldest old, also needs to be acknowledged.

Hence, there is an urgent need tackle loneliness in old age, reinforce social prescribing and address the barriers that prevent older adults in fully participating in the society.
